# Building a supply chain approach for an improved laboratory sample referral network in the Dominican Republic

**DOI:** 10.1186/2052-3211-7-S1-P4

**Published:** 2014-12-17

**Authors:** Alan George, Claudia Valdez, Martha Herrera, Edgar Barillas

**Affiliations:** 1 Management Sciences for Health, Systems for Improved Access to Pharmaceuticals and Services (MSH/SIAPS), Santo Domingo, Dominican Republic

## Background

Systems for Improved Access to Pharmaceuticals and Services (SIAPS) seeks to strengthen pharmaceutical systems by working with local partners in 20+ countries to develop and improve service delivery, human resources, health financing and information systems. At the request of the Dominican Republic Ministry of Health, SIAPS conducted a baseline study to identify key barriers affecting the timely diagnosis and treatment of HIV/AIDS and tuberculosis patients. To this end, an assessment of the country’s SCM of laboratory samples and test results was conducted.

## Method

The baseline study consisted of conducting in-person interviews with HIV/AIDS and tuberculosis program leaders, and a quantitative-qualitative study including data collection from 120+ health establishments in all nine regions of the country, including the National Reference Laboratory, where the majority of HIV/AIDS testing occurs and MOH administrative offices. Local personnel were trained in surveying to collect data regarding sample quality, turnaround times, and means of transportation. The data was analysed and interpreted by the lead researcher.

## Results

Key study findings indicate a lack of supply chain awareness across the laboratory samples network. Personnel reported a single focus from the clinical perspective. Varying vertical program-specific networks demonstrate an inefficient use of funds as well as physical and human resources. Based on these findings, SIAPS is supporting the design of a more efficient system by: designing a new transport flow for laboratory samples at the national level, creating supply chain key performance indicators for system measurement and continuous improvement, updating and documenting standard SCM Operating Procedures (SOPs), training of key personnel on SOPs.

## Discussion

A lack of SCM knowledge among health personnel in the Dominican Republic is a key contributing factor to the current limitations within the logistics system, which is composed of several vertical programs that work independently. The core of SCM is to have all involved working towards the same objective while making efficient use of available resources. Capacity building is needed to create a top-down laboratory sample referral system, where each level operates as a vital link in a unified supply chain. Training of local personnel will result in improved service delivery, ultimately translating to improved diagnosis and timely treatment of at risk populations.

## Lessons learned

As evidenced by this intervention, SCM is applicable to the referral network for laboratory samples. Personnel involved with the preparation, transportation, and reception of laboratory samples must be trained to have a supply chain orientation in order to understand and implement best practices.

